# Biomolecular Basis of Cellular Consciousness via Subcellular Nanobrains

**DOI:** 10.3390/ijms22052545

**Published:** 2021-03-03

**Authors:** František Baluška, William B. Miller, Arthur S. Reber

**Affiliations:** 1 Institute of Cellular and Molecular Botany, University of Bonn, 53115 Bonn, Germany; 2 Paradise Valley, Arizona, AZ 85253, USA; wbmiller1@cox.net; 3 Department of Psychology, University of British Columbia, Vancouver, BC V6T 1Z4, Canada; areber@brooklyn.cuny.edu

**Keywords:** actin, cell, cell biology, cytoskeleton, consciousness, eukaryotes, excitability, membranes, sentience, symbiosis

## Abstract

Cells emerged at the very beginning of life on Earth and, in fact, are coterminous with life. They are enclosed within an excitable plasma membrane, which defines the outside and inside domains via their specific biophysical properties. Unicellular organisms, such as diverse protists and algae, still live a cellular life. However, fungi, plants, and animals evolved a multicellular existence. Recently, we have developed the cellular basis of consciousness (CBC) model, which proposes that all biological awareness, sentience and consciousness are grounded in general cell biology. Here we discuss the biomolecular structures and processes that allow for and maintain this cellular consciousness from an evolutionary perspective.

**Motto: Within their insulating membranes, cells can establish order …they display a sense of purpose. Nurse, P. What is Life? (2020)**

## 1. Cellular Nature of Life

Nobel Prize winner Sir Paul Nurse in his latest book, *What is Life*? laments that we are underestimating cells [1]. He notes that every cell is a living entity endowed with all the properties that characterize living organisms. Unfortunately, the original form of Cell Theory, as postulated more than a hundred years ago, is plagued with several conceptual problems [2,3,4,5]. It is often ignored or forgotten that eukaryotic cells can, in fact, be viewed as multicellular ecosystems, in effect “cells within cells” [6,7]. Their organelles, such as mitochondria and plastids, are semi-autonomous endosymbiotic cells [8,9,10,11,12,13]. The symbiotic origin of the nucleus is emerging as a highly plausible scenario [9,14,15,16,17]. Cell theory has been an important concept unifying the whole of biology and has played a central role in our understanding of life [1,18,19,20]. However, several aspects of the theory require attention and amendment. A model based on biomolecular mechanisms of cellular consciousness [14,21,22] is a prime candidate in this respect, as it holds the key to a better understanding of life [22,23].

In addition, all multicellular organisms must recapitulate from a single-cellular form [17]. From both phylogenic and ontogenic perspectives, blue whales, humans, or sequoia trees, are all unicellular at the beginning of life. Even single bacterial or archaeal cells are endowed with life-specific characteristics and features to such an extent as to be properly deemed as having a basal form of proto-consciousness as well as intentional and cognitive capacities [24,25,26]. On the other hand, viruses are non-living entities outside of cells; they initiate life-like processes culminating in their replication immediately after entering their host cell. In order to replicate, viruses need a cellular environment. The whole cellular evolution, both prokaryotic and eukaryotic, is shaped by viral infections. The current COVID-19 pandemic makes this dramatically clear.

## 2. Chimeric Nature of the Eukaryotic Cell

In addition to prokaryotic bacteria and archaea, eukaryotic protozoa are considered to represent unicellular organisms. However, protozoa, as well as all other eukaryotic cells, are complex cells that evolved through endosymbiosis when one cell (typically bacterial, forming mitochondria and plastids) is incorporated by a host cell. It might well be that other cellular organelles are also of endosymbiotic nature. The difficulty is that over geological time, large amounts of DNA can be lost, as in the case of the highly reduced nuclei known as nucleomorphs, in which almost all of the DNA is transferred to the host cell nuclei [27,28]. Similar processes reduced the genome complexity of plastids and mitochondria during their endosymbiont-to-organelle transition for plastids see [29]. Recently, we [15,16,17] discussed the endosymbiotic origin of the eukaryotic nucleus that occurred when a host cell enclosed and endogenized a guest cell of apparent archaeal origin. In this proposal, all the host cell DNA is transferred to the guest cell, which is transformed into the eukaryotic nucleus [17]. In fact, one cannot exclude a putative endosymbiotic origin of several other organelles, such as endoplasmic reticulum, peroxisomes, centrosomes/centrioles and cilia/flagella [8,14,30,31]. In cellular evolution, cell–cell merging and endosymbiosis is an ancient and successful strategy, representing a fundamental feature and can also be seen in instances of the secondary and tertiary endosymbiotic events in algae [27,28,29,32,33]. In endosymbiosis, tinkering rather than whole-scale re-engineering is obvious [34] when large structures are continuously rearranged and recombined after cellular mergings of the formerly independent unicellular organisms [35,36].

## 3. Structures and Processes Behind Cellular Consciousness—Evolution of Chimeric Consciousness of Eukaryotic Cell

Lynn Margulis was one of the first scientists to seriously discuss the evolutionary origin of cellular consciousness and argued that prokaryotic cells that merged to form chimeric eukaryotic cells had their own prokaryotic-specific sentience [14]. In her view, the original prokaryotic cells had a “protoconsciousess”, and the two merged cells generated a supracellular consciousness. We develop this below from the perspective of the actin- and tubulin-based cytoskeletal elements where the host cell is proposed as a large archaea cell based on the actin cytoskeleton, while the small motile guest cell is based on the tubulin cytoskeleton supported by the centrosome and basal bodies/centrioles that animate eukaryotic flagella [11,15,16,17].

We recently discussed the biological foundations of cellular consciousness based on how an excitable plasma membrane, densely populated with so-called biological Maxwell demons, such as sensors, receptors, ion channels, transporters, and ATPases, can generate a senomic cellular field [22,23,37,38,39]. In the evolutionary origins of the eukaryotic cell (Box 1), both the large, actin-based host cell and the smaller guest cell, which relied on the tubulin-based cytoskeleton, were proposed to be ancient archaea [15,16,17]. This may allow the merging of their fields to generate the new stronger and senomic field of an emergent eukaryotic cell. In addition to the excitable plasma membrane and membranes of recycling vesicles, other cellular structures that are capable of contributing to the cellular fields are the large, bundled, vibrating elements of the cytoskeleton (Box 2), such as F-actin [40,41,42,43] and microtubules [44,45,46,47]. Both excitable plasma membrane and cytoskeletal elements have been proposed to generate proto-consciousness of individual eukaryotic cells [22,48].

Box 1 **History of Cellular Evolution: From the Prokaryotic Proto-Consciousness to the Eukaryotic Chimeric Consciousness.**

1/Emergence of the Last Universal Cell Ancestor (LUCA) from Proto-Cells. Fossil records and molecular clocks estimation about 4.3–3.5 billion years ago.

2/Emergence of Ancient Prokaryotic Cells and First Eukaryotic Common Ancestor (FECA). Fossil records and molecular clocks estimation about 1.8 billion years ago.

3/Emergence of the Last Eukaryotic Common Ancestor (LECA). Fossil records and molecular clocks estimation about 1.1 billion years ago.

4/Emergence n of the Eukaryotic Cell.

5/Emergence of Multicellular Eukaryotic Organisms. Fossil records and molecular clocks estimation about 0.8 billion years ago.

Box 2 **Subcellular Structures Supporting Cellular Conscioussness.**

1/Excitable Plasma Membrane

2/Plasma Membrane Based Endosomes

3/Endomembranes—Anterograde (Endoplasmic Reticulum, Golgi Apparatus)

4/Endomembranes—Retrograde (Endosomes, Trans-Golgi Networks)

5/Endomembranes—Organellar

6/Cytoskeleton—Actin Filaments and Myosis

7/Cytoskeleton—Microtubules, Kinesins and Dyneins

8/Cytoskeleton—Centrioles, Centrins and Centrosomes

9/Rotary ATPases at the Plasma Membrane and Endomembranes

10/Receptors and Sensors of the Plasma Membrane and Endomembranes

11/Ion Channels and Transporters of the Plasma Membrane and Endomembranes

Vibrations of excitable polymers contribute to the intracellular electromagnetic fields and can be expected to interact with the field emanating from the excitable plasma membrane. As microtubules act as memristors, as combinations of memory and electromagnetic resistance [49], they are well suited to faithfully decode the cellular senomic fields and to act accordingly. Furthermore, microtubules are structurally linked to both the actin filaments as well as the plasma membrane; they are perfectly suited to generate subcellular bioelectric circuits [49,50].

## 4. Structures and Processes behind Cellular Consciousness—Two Types of Nanobrains Generating Consciousness of Eukaryotic Cell

**Figure 1:**
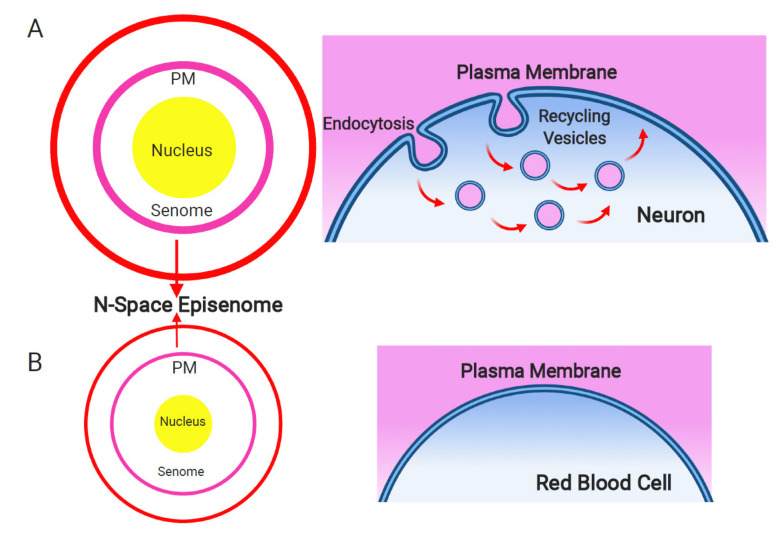
Plasma membrane and endosomal recycling vesicles-based nanobrain. Schematic depiction of the senome and the N-space episenome in two contrasting cells of multicellular organisms. (**A**) In the neurons and neuron-like cells, highly active endocytosis and endocytic vesicle recycling results in hypertrophied senome (lilac circle) and N-space episenome (red circle). Such cells are well-informed about their environment and are active in cell–cell communication via their plasma membrane-based nanobrains. (**B**) In the example of mature red blood cells, there are only minimal activities of endocytosis and endosomal vesicle recycling. Such cells have shrunk their senomes (lilac circle) and N-space episenomes (red circle) based nanobrains. They are socially isolated, with minimal cell–cell communication and highly reduced cellular sensory apparatus.

Two ancient cells merging into one resulted in the generation of supracellular chimeric consciousness having four different excitable sources: two plasma membranes, F-actin, and microtubules. The plasma membrane of the host cells, associated with the actin cytoskeleton, produced the senomic fields of contemporary chimeric eukaryotic cells. The guest cell transformed into the eukaryotic nucleus with the centrosome associated with centriole and organizing perinuclear microtubules [51]. The plasma membrane and the nuclear envelope/centrosome/microtubules complex can be viewed as two different cellular nanobrains, the origin, of which can be traced back to the two ancient cells, which merged together, forming the first eukaryotic cell [15,16,17]. Vibrations of F-actin and microtubules contribute significantly to the cellular electromagnetic field [45,46]. As microtubules act both as intracellular nanowires and memristors, they are perfectly suited for the nanobrain roles of the centrosomes/nuclear envelopes, complementing the principle nanobrain of the eukaryotic cell represented by the excitable plasma membrane (Figure 1, Box 1 and Box 2) inherently linked to the actin cytoskeleton.

## 5. Plasma Membrane as Primary Nanobrain

**Figure 2:**
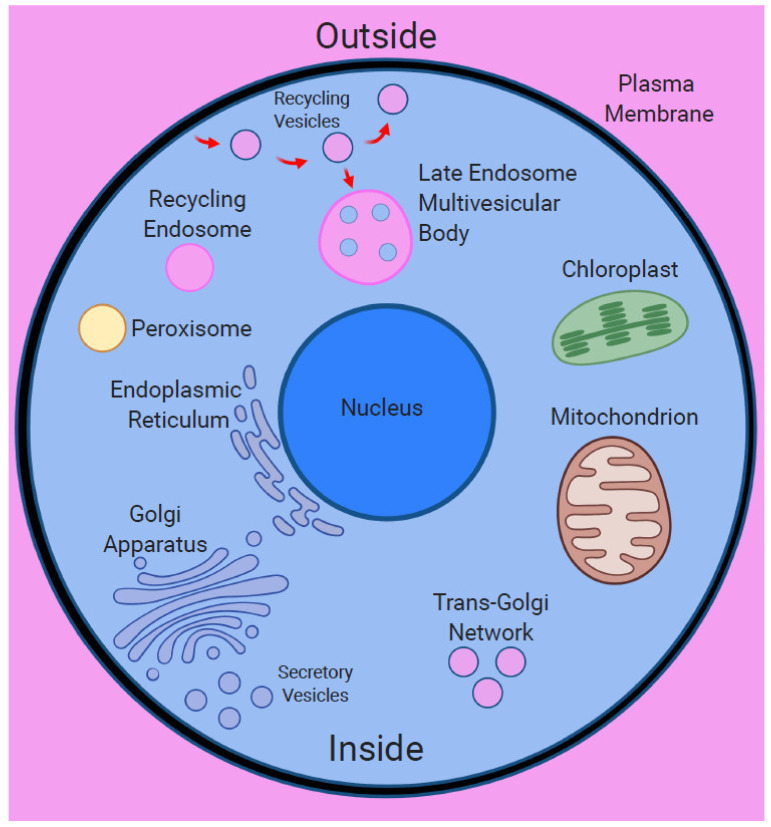
Surfaces within surfaces—endomembrane system of the eukaryotic cell. The eukaryotic cell has chimeric nature due to the endosymbiotic origin of its major organelles. The plasma membrane encloses diverse organelles as well as endocytic vesicles and endosomes, representing the surfaces within surfaces situation. Importantly, endocytic vesicles enclose portions of the extracellular space, representing unique *outside within inside* situation.

Based on the foregoing, it is proposed that the plasma membrane acts as the primary cellular nanobrain. It not only shelters the inside of the cell, but it also provides cells with all relevant sensory information via numerous sensors and receptors that densely populate the plasma membrane. Excited sensors and receptors, as well as rotary protein complexes, such as ATPases, which handle the energy flow across excitable membranes [52], act together as biological Maxwell demons [53,54,55]. These are central proteins for any living cells as they provide not only critical information but also mediate the handling of energy. The size of the plasma membrane can be expanded via the invagination of tubules as well as via endosomal vesicles generated by the plasma membrane. Endosomal vesicles enclose microspace topologically belonging to the extracellular space via membranes derived from the plasma membrane (Figure 1). This allows the expansion of the plasma membrane and amplification of the senomic field of the cell. From the biosemiotics perspective, the topology of surfaces within surfaces (Figure 2) plays a central role in the origin of agency and life [56]. In accordance with this concept, brain neurons and plant root cells specialized for cell–cell communication and brain-like activities are characteristics of recycling endosomal vesicles known as recycling (synaptic) vesicles [57,58]. In other words, the more endosomal/synaptic vesicles a cell is producing and the faster these vesicles recycle at the plasma membrane, the more robustly the cellular nanobrain develops, and the cell becomes more effectively informed about its environment (Figure 1). This plasma membrane-based nanobrain is acting at the intracellular-extracellular interface [59,60] and controls [61] the secondary cell body/energide nanobrain based on centrosomes and microtubules.

## 6. Centrosomes and Nuclear Surfaces as Cell Body/Energide Nanobrains

Cell bodies/energides control cytoplasmic space through radiating perinuclear microtubules [10,11,12,15,16,17,51]. These are organized by centrosomes/centrioles, which act as microelectronic choreographers of cells [5,17,62,63,64]. In both syncytial and coenocytic cells, the entire nuclear surface acts as centrosomal-like structures [10,12,51]. Both the centrosome and the nuclear surface can be considered as the second nanobrain of the eukaryotic cell, whose evolutionary origin can be traced back to the symbiotic origin of the eukaryotic cell [17]. This second cell nanobrain descended from the ancient guest cell [11,15,16,17] is central for cell polarity, cell division and cell movements [62,65,66,67]. In the polyenergide cells, such as syncytia and coenocytes, one plasma membrane nanobrain controls numerous cell body/energide nanobrains [10,12]. For example, up to several thousand nuclei were found to coexist in one large cytoplasm in an arbuscular mycorrhizal fungus [68], when some 35,000 nuclei carrying different genomes [69,70] were identified in a single fungal spore [68,71].

## 7. Nano-Intentionality and Nano-Mind from Eukaryotic Nanobrains

Nanobrains are behind the phenomenon of nano-intentionality [24], which is based on the fact that structural (phenotypic) plasticity is inherent not only to cells but is expressed in individual biomolecules. Cells continuously rearrange their molecules according to their actual sensory experiences mediated via senomic fields [37,38,39]. Senomic fields *animate* cellular biomolecules not only through biotensengrity [72,73,74] but also by electrical, magnetic, acoustic, and photonic and Lorentz forces, which permeate the cellular interior [75,76,77,78,79], continuously effecting changing conformations of all cellular biomolecules. Senomic nano-mind generated via cellular nanobrains allows a scale-free cognition to generate the *self* [37,48,80,81]. This cellular *self* is capable of obtaining meaningful content of the sensory information relevant for adaptation and survival [14,21,22,23,48,82,83,84]. In other words, the senomic *self* is proposed to allow the establishment of cellular purposiveness, allowing even unicellular organisms to be sentient and display cellular proto-consciousness [48,85]. That purposive agency is directed to the maintenance of cellular homeostatic equipoise in defense of that instantiated self [86]. Cellular proto-consciousness can thus explain the baffling abilities of unicellular organisms to act as intelligent organisms [82,87,88,89].

## 8. The N-Space Episenome as an Informational Matrix for Supra-Cellular Consciousness

The instantiation of consciousness was the induction of the living state. Necessarily then, all conscious life depends on the reception, assessment, and communication of information [23,90]. In any behavior, obligatory reception and assessment of information precede any deliberate communication, deployment of bioactive molecules, or energetic outputs. Therefore, the cellular appraisal of information is foundational to cellular consciousness and self-identity [86]. However, in the self-referential frame, any assessment of information is a measurement [38,39]. Self-referential cells are cognitive and must actively evaluate sensory information to sustain their homeostatic equipoise [21,22,23,83,91,92,93,94,95,96,97,98,99,100]. Cognitive cells must measure since their sensory information is imprecise. All biological information is clearly ambiguous [23,101,102,103] for two primary reasons. The first is based on thermodynamic requirements. In the self-referential frame, any assessment of sensory information requires work. Under the Second Law of Thermodynamics, work can never be converted with 100% efficiency. Second, all biological information available to cells must travel through varieties of media and across membranes. This transit degrades the validity of any initial source of sensory information due to time delays and engendered noise [90]. In considering the nature of sensory information, Bateson had astutely noted that biological information could be defined as “a difference, which makes a difference”, from which self-produced self-referential measurements can be made [103]. It follows from this precondition that all cells must have an attachment to an informational matrix as a set of essential reference points that enable each cell to purposively measure its homeostatic equipoise as a non-equilibrium state versus sequential environmental impacts [26]. This individualized cellular information field matrix represents all sources of information available to a cell, from which any “differences” essential to homeostasis can be ascertained.

In the cellular basis of consciousness (CBC), all cells are self-referential “knowing” problem-solving entities [21,22,23]. As cognitive agents, each cell has its own individualized information field through which it attaches to space–time information [23,37,38,39,104]. Crucially, it is this information matrix that impacts the cellular senome as the summation of the entire bioactive sensory apparatus of a cell (receptors, membranes, ion channels, the cytoskeleton, gap junctions) at any given moment [37]. This senomic assessment of information for any sentient cell is an absolute requirement for any connection between environmental inputs, the cellular genome, an intercalating epigenome and an expressive proteome [37,39].

It is the senome of any cell, acting as a sensory organ (Figure 1), that interlinks a cellular information field (the summation of all informational inputs) with the genome and epigenome through the plasma membrane and the cytoskeleton to participate in cellular problem-solving and cell–cell communication. In all multicellular organisms, the senome of any cell, and its attendant information field, intersects with the senomes of other cells as an aggregate senomic organization. This then becomes a composite multicellular informational matrix comprised of individual cellular informational matrices that overlap into a higher-order aggregate as an N-space episenome [38,39]. As such, it constitutes an overlapping supra-cellular aggregation of all the conscious individual cellular senomic responses to environmental inputs. In this manner, it functions as a shared measuring platform. There can be no doubt of its necessity. Collaborative life enables the sharing of resources, cooperative metabolic responses to stress, and coordinated reactions. Such a level of entwining cooperation mandates that a cohesive informational matrix is available at scale to permit coordination of the separable consciousness of each individual cellular participant to effect the type of supra-cellular consciousness that all holobionts represent. Simply put, coordinate life requires concordant measurements. In addition, consciousness at the level of any individual cell must find its reiterative manifestation as a functional aggregate supracellular consciousness to sustain holobionts in their confrontations with environmental stress [39].

The N-space episenome represents a whole-cell informational field projection. Although not itself material, it is nonetheless real as it has some correspondence with physical materiality through two means. In cognitive systems, information can be deemed physical since it directly relates to physical degrees of freedom [105]. The second is its straightforward link between environmental informational cues, the receptive and analytical senomic apparatus of the cell, the genome, and the proteome that all become linked material biological deployment. Therefore, the N-space episenome acts as an informational matrix that connects and coordinates the cellular senome, as the summation of the cellular sensory mechanisms, with the genome and epigenome [37,38]. In that regard, the N-space episenome acts as a reciprocating system allowing sentient handling and integration of sensory information [39].

It also has been proposed that this same N-space episenome represents a pre-existing heritable developmental information space architectural template for biological development and morphogenesis [38]. This overarching cellular architecture represents the direct pathway of supra-cellular deployment of senomic cellular resources upon which development depends. This is quite directly a necessity. All multicellular eukaryotes are holobionts as vast assemblages of differentiated eukaryotic cells and an essential partnering multispecies cellular microbiome with its own trillion of cells. Two requirements follow to successfully sustain this type of collective life. There must be some type of shared platform for measuring environmental stresses, and there also must be effective consonant communication among each of the “conscious” constituent cells.

At the level of entire holobionts, a heritable concordant means of assessing information in a self-similar manner in each successive generation is a default requirement. If it were otherwise, then there would be an inevitable skewing drift that would undermine any type of interconnected and reproducible supra-cellular consciousness that all organisms actually do exemplify. The N-space episenome enables aggregate supracellular consciousness by acting concurrently at two overlapping levels. It connects each cell to its individual information field as its individualized form of supracellular consciousness. In addition, it reiterates as a confederated platform for consonant senomic measurement among the diversity of cells that constitute holobionts to sustain those self-similar patterns of organismal supracellular consciousness that characterize all living forms.

## 9. Supra-Cellular Consciousness (Organismal Experience) Affects Cellular Structures

The biophysical nature of cellular consciousness is obvious from the fact that organismal sensory experiences feedback on diverse subcellular structures, including the F-actin-based neuronal processes known as dendritic spines [106,107,108]. These dynamic structures may be acting as receivers of both synaptic and senomic signals. They are known to be central for human and animal cognition via synaptic plasticity. Intriguingly, anesthetic isoflurane blocks F-actin-based motility and re-arrangements of these neuronal dendritic spines [108]. Moreover, sensory experiences control not only the neuronal wiring in the cerebral cortex but also exert control over the nuclear chromatin architecture in cerebellum neurons [109,110,111,112]. Even the minute nematode *Caenorhabditis elegans*, with a mere three hundred neurons, is capable of modifying cellular structures and physiological processes by recalling past aversive experiences implicating a *mind over matter* situation [113,114]. One of the functions of cellular consciousness is to assess the Implicate Order through a correct contextual interpretation of the sensory order [115,116]. In multicellular organisms, we can expect several levels of consciousness, starting with organelles of symbiotic origins, cells, tissues, organs and finally, the whole-organism level. Obviously, our organismal consciousness has no direct access to these lower levels of consciousness. This is an evolutionary safeguard for the multicellular organism, agency, which must focus on the higher-level tasks relevant for its survival and leave other tasks for the lower-levels of consciousness of organs, tissues, and cells. We would not be able to act as unitary organisms if we were aware of the lower levels of consciousness.

## 10. Life Is Electric: Bioelectric and Biomagnetic Nature of Life Processes

There are numerous definitions of life; the currently accepted view is that gene expression and DNA organization represent the foundations of life phenomena. However, it is rather obvious that DNA outside of cells is inactive, an inert macromolecule, which needs the support of numerous other macromolecules, especially proteins, to be functional in living organisms. In order to initiate and sustain life processes, excitable biomembranes populated densely with diverse ion channels, transporters, as well as receptors and sensors, are essential [53,59,117,118]. It is clear from the cyanobacterial invention of photosynthesis, a process generating the organic substances from inorganic ones, that photon-induced excitations of light-sensitive proteins release electrons, which then move via dedicated protein–protein complexes (donors and acceptors of electrons) of the photosynthetic apparatus [119,120]. Similar phenomena, based on moving electrons, are driving aerobic respiration in mitochondria [117,121,122,123]. Bioelectricity of membranes is controlled for the cellular handling of energy to support the life processes, and this bioenergetics is behind the emergence of mind and cognition [124,125,126]. Cellular bioenergetics was initiated by the discovery of vectorial chemistry by Peter Mitchell [127,128,129,130]. His chemiosmotic theory, which is central for our understanding of the bioelectric nature of bioenergetics, met substantial initial resistance. Peter Mitchell was a scientific dissident and financed his studies by himself [130]. With his discoveries, the early view of the bioelectric nature of life initially proposed by Luigi Galvani and Alexander von Humboldt [131,132] is now moving back to the center of biological sciences [80,118,133,134,135,136,137].

Unfortunately, contemporary biological and psychological sciences are locked in a Cartesian trap largely due to Descartes’ mind-body dualism. Currently, all biological sciences are dominated by deterministic machine-like mechanistic views and concepts [138,139,140]. The central dogma of molecular biology holds that DNA-based code instructs the formation of proteins and fates of cells [141,142]. However, more than sixty years ago, Albert Szent-Györgyi and his coworkers made it clear that the Cartesian metaphor (organism as machine) is not valid for the life processes, which are rather based on bioelectronics, macromolecular excitations, charge transfers, and electronic features of biomolecules [143,144,145,146,147,148,149,150]. As Szent-Györgyi noted in 1968. “While genetics, the conservation and transmission of the genetic code, is dominated by strict steric relations, the understanding of vital functions and the underlying energy transformation demand a more dynamic outlook on the electronic level.” [149]. Bioelectricity and biomagnetism of living cells generate dynamic biophysical fields, which underlie the unique features and properties of living systems [37,38,39,44,45,48,76,77,80], acting as sentient organisms [21,22,23,24,48].

## 11. Membranes and Proteins as Bioelectric Devices—Proteins *Dance* to Senomic *Tunes*

One of the most important messages from Szent-Györgyi’s research is that life is based on electron transport chains starting with the photosynthetic pigments excited with photons arriving from the Sun [119,120,151,152]. Similarly, excited and moveable electrons also underlie mitochondrial respiration processes [121,122,123,152,153]. Both the photosynthetic and respiratory super-complexes rely on membranes allowing their assembly and function. In addition, the plasma membrane and derived endosomal/vesicular membranes support transmembrane electron transport as an essential feature of any eukaryotic cell [154,155,156,157,158,159,160,161,162]. Importantly, ultrafast and abundant electron transfers occur within proteins [163]. Thus, not only the sequence of amino acids but especially these electrostatic forces control post-translational protein folding [164,165,166]. The central dogma of molecular biology is missing this biological reality as the three-dimensional conformations of proteins are not dictated solely by the information encoded in DNA sequences [141,142], but rather through the bioelectric properties of proteins and their subcellular physicochemical senomic environment, including special properties of water interacting with diverse cellular surfaces. Moreover, any biological structure acts as information relevant for biocommunication, which implies that the basic life processes have fundamentally cognitive features [22,23,24,124,125,126,140,167,168,169,170,171,172,173,174,175,176]. In a poetic language, proteins are dancing to senomic tunes within the cellular senomic environment.

## 12. Cells as Unitary Organisms: From Mechanicism to Organicism

Szent-Györgyi also stated that the living cell is a system driven by energy flows [149]. Cell integrity requires excitable lipid-based membranes defining the inside (living system) from the outside (non-living system) (Figure 1 and Figure 2), which is the cellular basis of the sentient subjectivity [21,22,23,24,48]. Life was unicellular for about two billion years; true multicellularity evolved in eukaryotes relatively recently. Nobel Prize winner Barbara McClintock raised two critical questions with respect to cells acting as organisms. First, what is the extent of knowledge a cell has of itself and second, how does a cell use this knowledge in a thoughtful manner when it is challenged [177,178]? Considering cells as the basic units of multicellular organisms enjoying and protecting their self-identities via their cellular sentience promotes fresh concepts that deepen our understanding of these organisms. It is expected to be very relevant for our understanding of the cellular basis of diverse diseases, especially of cancer and neurodegenerative diseases.

## 13. Outlook

Mechanistic concepts have long dominated thinking in biology [179,180]. This world view has encumbered the biological sciences and prevented a full integration of the true nature of living cells and their attendant biological consciousness into a renewed evolutionary framework. Cells not only generate their own electromagnetic fields but are highly sensitive to extracellular electromagnetic fields [76,77,181,182,183,184,185,186]. It has been recently reported [186] that action potentials traveling along vascular bundles of carnivorous Venus flytrap plants induce biomagnetic fields. In fact, cellular bioelectricity has a significant role in the control of development, morphogenesis, and regeneration at all levels of biological complexity [80,81,133,136,187]. Already bacteria use bioelectricity both to establish memories and for biocommunication [188,189] that energizes their own prokaryotic-specific nanobrains [169,188,189,190,191,192,193,194]. During cellular evolution, additional functions resulted in the more complex and sophisticated cellular nanobrains of eukaryotic cells (Box 1). Since organelles of eukaryotic cells, such as mitochondria and plastids, are of bacterial origin, one can expect further discoveries in our understanding of cellular nanobrains (Box 2). These advances can proceed with the perspective that the eukaryotic cell is a cognitive and intentional supracellular consortium [10,11,14,15,16,17,21,22,23,48]; integrated through ancient proto-signaling networks based on electrostatic forces and reactive electrophilic redox species [195,196,197,198,199,200], dynamic cytoskeleton, and subcellular communication across organellar synapses [201].

## Data Availability

Not applicable.
